# Determination of total phenolics, flavonoids, and testing of antioxidant and antibacterial activities of red ginger (*Zingiber officinale* var. Rubrum)

**DOI:** 10.5455/javar.2024.k755

**Published:** 2024-03-19

**Authors:** Ucop Haroen, Syafwan Syafwan, Kiki Kurniawan, Agus Budiansyah

**Affiliations:** 1 Faculty of Animal Science, Jambi University, Sumatra, Indonesia; 2 Research Center for Vaccine and Drugs Development, National Research and Innovation Agency, Cibinong, Indonesia

**Keywords:** *Zingiber officinale var. Rubrum*, antioxidant, antibacterial, MIC, MBC

## Abstract

**Objective:**

The purpose of this study was to select the active fraction of red ginger *(Zingiber officinale var. Rubrum)* for its antioxidant and antibacterial activities against *Staphylococcus aureus* (AMC 6934), *Bacillus subtilis* (AMC 7923), *Pseudomonas aeruginosa* (AMC 8973), and *Escherichia coli* (AMC 5761).

**Materials and Methods:**

A total of 2 kg of dry red ginger rhizome powder was macerated in stages with different levels of solvent polarity to extract the chemical composition within the red ginger powder sample. The extraction process begins with a non-polar solvent (n-hexane) by soaking the red ginger powder sample for 3 × 24 h.

**Results:**

The red ginger extract fractionated with methanol produced alkaloids, phenolics, flavonoids, and coumarins, while the fractionation using n-hexane produced alkaloids and triterpenoids only. The fractionation with ethyl acetate produced alkaloids, phenolics, flavonoids, triterpenoids, saponins, and coumarins. The antioxidant activity test was 49.261 mg/l for the ethyl acetate fraction, 146.648 mg/l for the methanol fraction, and 300.865 mg/l for the n-hexane fraction.

**Conclusion:**

The ethyl acetate fraction was effectively powerful in inhibiting the growth of Gram-positive and Gram-negative bacteria. All fractions had moderate antibacterial activity; however, the performance of ethyl acetate in the red ginger extract was better than that of methanol and n-hexane.

## Introduction

The provision of additional feed additives in animal feed, such as antibiotics, enzymes, coccidiostats, antioxidants, antifungals, prebiotics, and organic acids, has been widely carried out to increase the shelf life of feed, increase animals’ growth, and increase feed consumption [1,2]. Despite the advantages, additional feed additives have negative impacts on those consuming the livestock products, as they are associated with the chemical residue within the livestock products, such as chicken meat and eggs, as well as resistance to pathogenic bacteria. Since January 1, 2006, the use of additional feed additives or synthetic feed additives is no longer permitted in the European Union and Asian countries to protect consumers from their negative effects. Recently, consumers have demanded nontoxic and healthy livestock products using natural feed ingredients [3].

Natural feed additives from plants or medicinal plants have captured people’s attention due to their massive number of secondary metabolites [4] and their beneficial effects. One of the many efficacious herbs or medicinal plants that is rich in secondary metabolites is red ginger *(Zingiber officinale var. Rubrum)*. Some secondary metabolites contained in red gingers are gingerols, shogaols, gingerdiols, and gingerdio, where those compounds have a pharmacological effect that is beneficial for the health of livestock, especially poultry [5]. Zhao et al. [5] stated that red gingers could also increase appetite and nutrient absorption in the digestive tract. All of this occurs due to the positive effect of secondary metabolites in red ginger, which can increase the secretion of digestive enzymes such as lipase, disaccharidase, and maltase. The secondary metabolites in red gingers are derivatives of phenolic ketones that can reduce cholesterol in poultry products, including cholesterol in eggs, and can reduce oxidation in animal feed stored for a long time. Red gingers are herbs that belong to the *Zingiberaceae* family, the genus *Zingiber*, and the species *Zingiber officinale Rubrum*. Red gingers are commonly discovered in Central Asian countries, especially China, India, Pakistan, and Indonesia. They are popular, and they have been exported worldwide. In addition, red ginger has been widely used as a raw material for certain diseases in traditional medicine [6].

The rhizome of red ginger has more benefits as compared to other kinds of ginger, especially in terms of the number of secondary metabolites and its biological activity, such as antioxidant and antibacterial [7]. Another auspicious impact of red ginger is not only that it is used for traditional medicine but is also beneficial for the physical preservation of poultry fields [8]. In addition to those compounds, red ginger also contains polyphenols and flavonoids, which function as antioxidants [9].

Herawati [10] revealed that red gingers contain sesquiterpene essential oils, which are prospective to diminish toxicity in livestock. The essential oil within red ginger significantly escalates the antibacterial activity of *Staphylococcus*, *Escherichia coli,* and *Salmonella* [11]. In previous research, the extraction of phytochemical compounds from red ginger rhizome was solely carried out using water or polar solvents [12], while extractions using other solvents such as ethyl acetate and n-hexane and their potential as antioxidants have not yet been widely reported.

In addition, previous studies on antibacterial activity tests were conducted using the disc method, whereas the test in this study was completed with the dilution (diffusion) method. This method was selected as it is the most appropriate to determine the minimum concentration required to suppress the development of pathogenic bacteria. Hence, an overall conclusion on the minimum concentration of red ginger extract needed as an antibacterial or its function to inhibit pathogenic bacteria could be gained.

This research aimed at determining the ability or power of the red ginger fraction *(Zingiber officinale var. Rubrum)* towards antioxidant and antibacterial activities in inhibiting the growth of pathogenic bacteria that frequently infect poultry, such as *E. coli* (AMC 5761), *Staphylococcus aureus* (AMC 6934), and *Pseudomonas aeruginosa* (AMC 8973), and also of non-pathogenic bacteria, namely *Bacillus subtilis* (AMC 7923). Therefore, the feed coming from red gingers *(Zingiber officinale var. Rubrum)* has the potential to be an additional feed component in poultry rations.

## Materials and Methods

### Research tools and materials

A total of 2 kg of fresh red gingers from Jambi were dried at room temperature and crushed to result in red ginger powder. The solvents acquired from Perseroan Terbatas (PT). Bratachem Indonesia was methanol, ethyl acetate, and n-hexane. Furthermore, standards of gallic acid, quercetin, and calcium chloride were obtained from PT, Merck Tbk. The 2,2-diphenyl-1-picryl-hydrazine-hydrate (DPPH) free radical and Whatman filter paper were obtained from PT, Sigma Aldrich.

### Red ginger sample preparation

This study used the maceration method, which was done gradually with different levels of the polarity of solvents, to extract the chemical compounds within the red ginger powder [13]. The extraction process was started with a non-polar solvent (n-hexane) by soaking red gingers for 72 h. It was then continued with a semi-polar solvent (ethyl acetate), which aimed to extract semi-polar compounds from the sample (red ginger powder) for 3 × 24 h. The next maceration process was also conducted for 72 h using a polar solvent, methanol.

### Secondary metabolite compound screening

Metabolite compounds were tested qualitatively following the methods written by Hossain et al. [14] and Kancherla et al. [15]. The alkaloid test was done using Mayer’s reagent and Dragendorff’s reagent. The flavonoid test was conducted according to the Shinoda test, and iron (III) chloride was utilized to test for phenolics. Furthermore, saponins were tested by adding concentrated hydrochloride (HCl) until the foam remained. Finally, the terpenoid or steroid test used the Liebermann–Burchard test.

### Antibacterial activity test

The antibacterial activity test in this study used the dilution method (diffusion). This method was selected since it was the most appropriate to determine the minimal inhibitory concentration (MIC) and the minimum bactericidal concentration (MBC). The use of diffusion was intended to verify the concentration of secondary metabolites that function as antibacterial agents in the agar plate. This method is a quantitative method for accurately determining secondary metabolite concentrations that inhibit the *in vitro* growth of bacteria [16].

### Bacterial culture test

*Escherichia coli* (AMC 5761) and *P. aeruginosa* (AMC 8973) can cause urinary tract infections. In addition to that, *E. coli* (AMC 5761) can induce pneumonia. More negative impacts were caused by *S. aureus* (AMC 6934), such as bone pain, joint pain, skin irritation, and boils. Last, *B. subtilis* (AMC 7923) can cause pneumonia and diarrhea.

### Inoculum preparation

A 0.5 McFarland standard was initially prepared for the bacterial inoculum. The bacteria cultured in nutrient agar were then transferred into an agar plate in a test tube and incubated for 24 h at 37°C. The bacterial colonies within the agar plate were diluted with a physiological saline solution, and its turbidity was determined by using the 0.5 McFarland standard, which is equal to 1 × 10^8^ CFU/ml [17].

### Antibacterial activity test using the disc method

The antibacterial activity test was done on each extract of red ginger that had been fractioned into three parts by using four types of bacteria, namely *E. coli* (AMC 5761), *P. aeruginosa* (AMC 8973), *S. aureus* (AMC 6934), and *B. subtilis* (AMC 7923). The positive control in this antibacterial activity test used a 200 ppm tetracycline antibiotic solution, while the negative control used 100% ethanol. The antibacterial activity test was completed using the disc method. The antibacterial activity of each tested fraction was determined by calculating the clear zone diameter produced around the disc after the test or experiment [18]. The antibacterial activity test of the three fractions of red ginger can be seen in Table 1.

**Table 1.** The antibacterial activity test of red ginger fraction.

The result of the antibacterial activity test of red ginger extract				
Sample	Diameter of clear zone (mm)			
	*E. coli*	*P. aeruginosa*	*B. subtilis*	*S. aureus*
Methanol	8.80	7.80	8.70	8.70
	8.70	7.70	8.80	8.90
	8.75	7.75	8.75	8.80
STDEV	0.05	0.05	0.05	0.10
Ethyl acetate	8.90	8.80	8.90	9.80
	8.80	9.70	8.80	8.70
	8.85	9.25	8.85	9.25
STDEV	0.05	0.45	0.05	0.55
n-hexane	7.80	7.80	7.70	7.70
	7.70	7.60	7.60	7.60
	7.75	7.70	7.65	7.65
STDEV	0.05	0.10	0.05	0.05
Control +	14.65	15.14	19.69	20.23
Control -	5.00	5.00	5.00	5.00

*Note:* The test was conducted at the Microbiology Laboratory, Vaccine and Drug Research Centre, National Research and Innovation Agency, 2022.

### MIC test

This test was accomplished to calculate the minimum concentration necessary for the red ginger extract to inhibit the growth of the tested bacteria. The microdilution method was used, and the result was decided by observing turbidimetry or turbidity visually [19]. Observation of the antibacterial activity test was completed by looking at the minimum concentration’s ability to inhibit the bacteria’s growth after 24 h of incubation.

### MBC test

Determining the MBC for each fraction of red ginger is the main emphasis of this research. The purpose of this study was to determine the ability of the red ginger fraction to inhibit the growth of the bacteria tested, namely *E. coli*, *P. aeruginosa*, *S. aureus,* and* B. subtilis*. The value of the MBC was gauged by observing the lowest concentration of the tested sample that did not show any bacterial growth on the inoculated agar microplates.

### Determining the total of phenolic

The concentration of phenol in the red ginger powder sample was ascertained by using a spectroscopic method, as explained by Khan et al. [20]. The reaction mixture was prepared by weighing 1 mg of the extract to be mixed with 1 ml of 10% Folin-Ciocalteu reagent dissolved in 13 ml of distilled water. Hereafter, 5 ml of a 7% Na_2_CO_3_ solution was added. The mixture was stirred until it resulted in a homogeneous mixture and stored in a dark room for 2 h. In the next stage, a blank solution was prepared by adding 0.5 ml of methanol, 2.5 ml of 10% Folin-Ciocalteu reagent, which was dissolved in water, and 2/5 ml of 7.5% Na_2_CO_3_ before it was stored at 45°C for 45 min. Subsequently, an absorbance measurement was carried out by using a spectrophotometer at λ_max_ = 765 nm. For the standard gallic acid solution, the same procedure was applied, and a calibration curve was made. The phenolic concentration obtained was based on the absorbance value measured on the spectrophotometer utilized. The phenolic concentration was obtained from the calibration curve, and the total phenolic content in the extract was expressed with the term gallic acid equivalent (mg GA/gm extract).

### Determining the total of flavonoid

The amount of flavonoid in the tested sample was calculated using the spectroscopic technique [21]. As much as 1 mg of the extract was dissolved in a mixture of 1 ml methanol and 1 ml of 2% AlCl_3_ solution in methanol before it was incubated at room temperature for one hour. Henceforth, the absorbance was determined using a spectrophotometer at λ_max_ = 415 nm. The incubated sample was then analyzed to obtain the absorbance value. A similar procedure was taken to determine the calibration curve of the quercetin standard solution and to form the calibration curve. Based on the absorbance value obtained from the instrument, the value of flavonoid concentration was read (mg/ml) on the calibration curve. Flavonoid levels contained in the test extracts were declared equivalent to standard quercetin (mg Qu/gm extract).

### Determining beta-carotene levels

The red ginger sample (*Zingiber officinale var. Rubrum*) was weighed for 50 gm before it was macerated with a mixture of n-hexane, acetone, and calcium chloride (CaCl_2_) with a ratio of 1:1:1. The mixture was then centrifuged at 3,000–5,000 rpm for 15 min, and the precipitation must be separated from the filtrate. The formed precipitate was washed with a saturated CaCl_2_ salt solution before being separated by filtrate. Hereafter, the precipitate was dried using a rotary evaporator at 40°C [22,23].

### The results of extraction by maceration

The crude extract obtained through a maceration process resulted in more methanol extract compared to the ethyl acetate or n-hexane extract. From this process, it can be inferred that methanol was a good solvent used to extract the components of secondary metabolites in the red ginger [24]. The extraction result of the red ginger powder using three types of solvent is shown in Table 2.

### Antioxidant activity evaluation

The capability of plant extracts to capture free radicals (DPPH) can be determined quantitatively. To begin with, the mother liquor of each extract was prepared to be dissolved in methanol to obtain a concentration of 1 mg/ml. Furthermore, a gradual dilution was carried out to gain concentrations of 10, 50, 100, and 200 mg/ml into the test solution whose concentration has been determined. Then, DPPH in methanol with a concentration of 1 mg/ml was added. Furthermore, the solution was stored in a dark place for 30 min at a room temperature of 25°C to reach optimum conditions. The absorbance measurement was carried out at a wavelength of 517 nm. As for the control solution, quercetin, which had been diluted in stages with various final concentrations of 5, 15, 25, and 50 mg/ml, was used. The percentage of inhibition was calculated using the formula in equation (1), where the value of IC_50-_ was determined from the graph of the percentage of inhibition versus the concentration of the sample using a non-linear calculation algorithm [20,21].

**Table 2.** The result of red ginger extractions from methanol, ethyl acetate, and n-hexane solvents.

Solvent	Total (gm)
Methanol	234.575
Ethyl acetate	128.287
n-hexane	106.288

*Note:* The result of red ginger extractions from methanol, ethyl acetate, and n-hexane solvent in the Microbiology laboratory, Vaccine and Drug Research Centre, National Research and Innovation Agency, 2022.

(%) Inhibition = [1-(As/A0)×100.] (1)

### Preparation of quercetin solution

In this research, the total antioxidant of the control positive from the extract of the red ginger sample was determined using a quercetin solution. The quercetin standard was made by putting 10 mg of quercetin crystals into a 100 ml volumetric flask, which was then dissolved in 5 ml of methanol. Once the quercetin crystals had completely dissolved, the volume of the solution was filled with ethanol or by adding 95 ml of ethanol. The 100 ppm quercetin mother liquor was then diluted in stages using a volumetric flask to obtain final concentrations of 5, 15, 25, and 50 ppm.

## Results and Discussion

### The results of extraction by maceration

The result of the crude extract obtained through the maceration process produced more methanol extract than ethyl acetate or n-hexane. In this maceration process, it can be concluded that methanol is good for extracting components of secondary metabolites in red gingers (*Zingiber officinale var. Rubrum*) [24]. Table 2 below shows the extraction results of red ginger powder from three types of solvents.

Overall, methanol has the best result in the extraction process using the maceration technique. This is reasonable because methanol is a universal solvent that can dissolve all types of polarity. From Table 2, it can be seen that methanol’s ability to extract secondary metabolites is better than the other two solvents. A total of 234.575 gm of concentrated methanol was extracted from 2 kg of red ginger powder. Meanwhile, the concentrated ethyl acetate and n-hexane fractions obtained were 128.287 and 106.288 gm, respectively.

### Secondary metabolite test of red ginger powder

From the secondary metabolite test conducted on the three fractions, which were obtained after the maceration process, the red ginger powder was discovered to contain a secondary metabolic compound, which can be seen in Table 3.

### Determining the total phenolic compound

To determine the total phenolic content of the methanol and ethyl acetate extracts of red ginger, the Folin-Ciocalteu reagent was used by equalizing the gallic acid concentration. The obtained value from the spectrophotometer was put into the regression equation (*y* = 0.0591*x* – 0.2823, *R*^2^ = 0.9869). The total phenol concentration obtained was substituted into the value of mg GA/extract, and it was determined that the total phenol concentration in the extract ranges from 28.4415 to 38.1712 mg GA/extract. The highest level of the phenolic compound was in the ethyl acetate fraction, with a concentration of 38.1712 mg GA/extract. For the methanol fraction, there was a decline in the phenolic compound within it at 33.6593 mg GA/extract. This amount of phenolic compound in the phenolic fraction was gained because the phenolic compound in the red ginger sample has semipolar properties [21].

In a study conducted by Lukiati et al. [25], the determination of phenolic concentration in ethanol and methanol extracts from red ginger rhizome resulted in 155.784 mg GA/extract and 132.541 mg GA/extract. This high amount of phenolics was obtained because it used crude ethanol and methanol extracts that had not been fractionated based on the polarity of the phenolic compounds. Therefore, all phenolic compounds will dissolve in both types of polar solvents. Moreover, in this study, the solubility of phenolic compounds was separated based on their polarity, giving a different total concentration for each extract. In addition, the phenolic compound in this study was more specific based on the polarity of the solvents. The total phenolic compounds within each extract are strongly related to the method of the extraction process. In this research, the extraction method used was a gradual, multilevel extraction process that started with a non-polar solvent. As a result, all groups of non-polar compounds would be initially extracted into non-polar solvents. The semi-polar compounds, however, would be perfectly extracted into the semi-polar solvents; in this study, ethyl acetate was used. The group of phenolic compounds and their by-products would be completely extracted in semi-polar solvents, thus enabling an increase in the total phenolic concentration in the ethyl acetate extract [26]. The total concentration of phenol in the ethyl acetate and methanol fractions is shown in Table 4.

### Determining the total of flavonoid

The determination of the total flavonoids from samples of the red ginger powder using the quercetin standard was carried out on fractions that contain flavonoids, as evidenced by the results of the secondary metabolites test. The fractions of flavonoid concentrations were determined by ethyl acetate and methanol. It was accomplished by using the spectrophotometer, where a regression curve was formed. Calculating the total flavonoid from each fraction tested, the absorbance value was substituted from the measurement into the quercetin standard regression curve (*y* = 0.0089*x* + 0.0264, *R*^2^ = 0.9813). The total amount of flavonoids expressed in Qu/extract. In this research, the total of flavonoids in the red ginger extract fractionated using three different solvents with different polarities reveals that the total of flavonoids in the ethyl acetate and methanol fractions was high, between 46.66 and 57.78 mg/gm It occurs owing to the nature of the flavonoid itself, which can be easily dissolved in semi-polar solvents (i.e., ethyl acetate) [27]. Lukiati et al. [25] stated that determining the level of flavonoids in the red ginger extracts concentrates on the level of solubility in two types of solvents with different polarities. In this research, it was discovered that the group of flavonoids dissolves into two types of polarities based on the number of substituent groups present in the core framework of the flavonoids [28]. The following Table 5 describes the total amount of flavonoids in the ethyl acetate and methanol fractions.

**Table 3.** The result of the secondary metabolite test on methanol, ethyl acetate, and n-hexane fractions.

No	Test parameter	Observation	Methanol fraction	Ethyl acetate fraction	n-hexane fraction
1	Alkaloid	Orange precipitate	Orange precipitate (√)	Orange precipitate (√)	Orange precipitate (√)
2	Phenolic	Dark purple	Purple solution (√)	Purple solution (√)	No purple solution (×)
3	Flavonoid	Red-orange-coloured solution	Red-coloured solution (√)	Red-orange-coloured solution (√)	No red-orange-coloured solution (×)
4	Triterpenoid	Red-purple-coloured solution	No red-purple-coloured solution (×)	Purple-coloured solution (√)	Purple-coloured solution (√)
5	Steroid	Green-coloured solution	No green-coloured solution (×)	No green-coloured solution (×)	No green-coloured solution (×)
6	Saponin	The foam does not disappear after the addition of concentrated HCl	Foam is formed after the addition of concentrated HCl (×)	Foam is formed after the addition of concentrated HCl (√)	Foam is not formed after the addition of concentrated HCl (√)
7	Coumarin	Blue-green fluorescence appears under the 365 nm UV lamp after it is sprayed with 2% NaOH	Blue fluorescence was identified after spraying with 2% NaOH (√)	Yellow-green fluorescence appears after spraying with 2% NaOH (√)	No fluorescence after 2% of NaOH was sprayed (×)

*Note:* Screening of the secondary metabolite was done in the Natural Material Chemistry Laboratory, Chemistry Research Centre, National Research and Innovation Agency, 2022.

**Table 4.** The total of phenolic compounds in the ethyl acetate and methanol fraction expressed equivalent to gallic acid (mg GA/extract).

Fractions	Absorbance	Phenolic levels (mg GA/extract)	Average + SD
Ethyl acetate	1.1246	38.12203	38.1712 ± 0.0695
	1.1275	38.22034	
Methanol	0.9983	33.84068	33.6593 ± 0.2564
	0.9876	33.47797	

*Note:* The determination of the total of phenolic in the ethyl acetate and methanol fractions was carried out in the Natural Material Chemistry Laboratory, Chemistry Research Centre, National Research and Innovation Agency, 2022.

### Determining the total antioxidant activity

Previous studies revealed that ethyl acetate and methanol fractions contain groups of polyphenolic compounds such as phenolics and flavonoids. Stanković et al. [21] and Lezoul et al. [13] declared that phenolics and flavonoids have antioxidant activity. This antioxidant is believed to be capable of inhibiting an oxidation process [29]. In this research, the antioxidant activity test used stable free radicals, such as DPPH solution, in a methanol solvent.

The antioxidant activity test was performed on three fractions obtained from the maceration process (methanol, ethyl acetate, and n-hexane). The test result showed that ethyl acetate has better activity in inhibiting free radicals, which was added to the reaction, as compared to methanol and n-hexane. It indicates the response given by polyphenolic compounds in the ethyl acetate fraction extract. The higher the amount of phenolic and flavonoid compounds in the extract, the better the extract’s response to obstructing the growth of free radicals [30]. This is proven by the ability of the ethyl acetate fraction to inhibit the growth of 50% free radicals (IC_50_) at a concentration of 49.261 g/ml. Moreover, moderate activity was shown by methanol in inhibiting the development of free radicals at 146.648 mg/l. The lowest activity was found in the n-hexane fraction (300.865 mg/l). This lower activity of the n-hexane fraction indicates that phenolics and flavonoids are insoluble in non-polar solvents, thus affecting the free radical scavenging activity produced by DPPH [31]. Another possible explanation is that the n-hexane fraction does not contain any groups of polyphenolic compounds (phenolics and flavonoids). The antioxidant activity of the n-hexane fraction is believed to occur due to the presence of beta-carotene in the fraction.

**Table 5.** The total flavonoids from ethyl acetate and methanol fractions of red gingers.

Fractions	Absorbance	Flavonoids levels (mg Qu/extract)	Average + SD
Ethyl acetate	0.2891	59.03	57.78 ± 1.7796
	0.2779	56.52	
Methanol	0.2397	47.93	45.66 ± 1.7955
	0.2284	45.39	

*Note:* The examination was done in the Natural Material Chemistry Laboratory, Chemistry Research Centre, National Research and Innovation Agency, 2022.

**Table 6.** Antioxidant activity of ethyl acetate fraction of red ginger.

Concentration	Absorbance	% Inhibition	IC_50_
200	0.1865	90.5898	49.2610
100	0.5637	71.5575	
50	0.9160	53.7817	
10	2.4510	32.1333	

*Note:* The examination to determine the antioxidant activity of the ethyl acetate fraction of red gingers was conducted in the Natural Material Chemistry Laboratory, Chemistry Research Centre, National Research and Innovation Agency, 2022.

**Figure 1.**
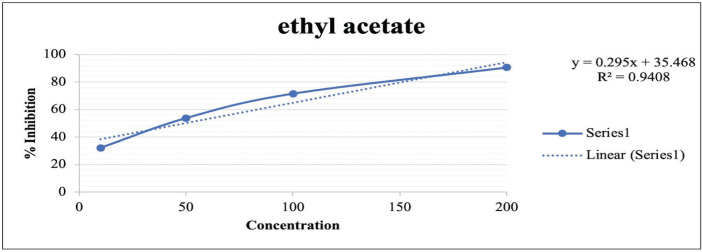
The relationship between the percentage of inhibition and the concentration of ethyl acetate fraction of red ginger.

The extract that has high antioxidant activity is significantly influenced by the high amount of polyphenolic compounds. The two groups of polyphenolic compounds (phenolic and flavonoids) are crucial in capturing or inhibiting free radical activity due to the presence of the hydroxyl group (–OH) at the core of these compounds. Based on many studies regarding herbal plants, it has been found that phenolics and flavonoids are directly responsible for the antioxidant activity of herbal plants [32–34]. Furthermore, the supplementation of ginger flour *(Zingiber officinale)* in the hen rations prominently increases the activity of antioxidant enzymes in serum and egg yolk. The value of antioxidant activity from the ethyl acetate fraction can be seen in Table 6, and the relationship between the percentage of inhibition and the concentration of the ethyl acetate fraction is illustrated in Figure 1.

### Determining the amount of beta-carotene

Isolation and determining the beta-carotene total of the red ginger sample are strongly influenced by the solvent used. The use of solvents must correspond to the physical properties of beta-carotene itself. In this study, the use of the non-polar solvent n-hexane considerably affects the extraction of beta-carotene. An explanation for this is the very high solubility of beta-carotene in the non-polar solvent [35]. To achieve the maximum result, acetone and saturated calcium chloride are added to increase the solubility of beta-carotene in organic solvents. The mixture of saturated calcium chloride and acetone in the n-hexane solvent will facilitate the extraction process of beta-carotene within the cell walls of red ginger plants. In addition, the use of saturated calcium chloride aims to precipitate the double chain found in beta-carotene. The extraction result was centrifuged to separate the precipitate from the beta-carotene dissolved in the organic solvents, a mixture of n-hexane and acetone [22].

From the research result, a bright orange-colored solution was found, and then the solvent was evaporated slowly to obtain an orange-colored precipitate weighing 0.3725 gm. Using the equation below, the total beta-carotene in the red ginger sample can be calculated. The value of beta-carotene obtained from this study was an approximate calculation done by calculating the percentage of beta-carotene that was successfully isolated using a mixture of n-hexane, acetone, and calcium chloride. Meanwhile, research on the beta-carotene isolation from carrot flour samples used a similar method, resulting in around 29.16 ppm beta-carotene [23].


$$\mathrm{beta}\;\mathrm{carotene}=\frac{0.3725\;\mathrm{gm}}{50\;\mathrm{gm}}\times 100\%=0.745$$

### Disc diffusion test of antibacterial activity

The disc diffusion test of antibacterial activity using the Whatman filter paper is a moderately effective method to determine the antibacterial activity of an extract. This antibacterial test used the diffusion method by varying the concentration of extract to observe the antibacterial activity of the tested extract on the inhibition of bacterial growth. The ability of an extract to obstruct bacterial growth is determined by the diameter of the inhibition zone produced after the test is completed [36]. In this study, four types of bacteria were used, consisting of two gram-positive bacteria, namely *S. aureus* and *B. subtilis*, and two gram-negative bacteria, namely *E. coli* and *P. aeruginosa*. This antibacterial activity test was carried out on red ginger extract, which has been simplified into three fractions, namely methanol, ethyl acetate, and n-hexane. The activity of each fraction was gauged by the ability of the inhibition zone produced from each fraction of extract. This is designated by the presence of a clear (inhibition) zone around the disc used. The antibacterial activity of red ginger extract can be seen in Table 1.

**Figure 2.**
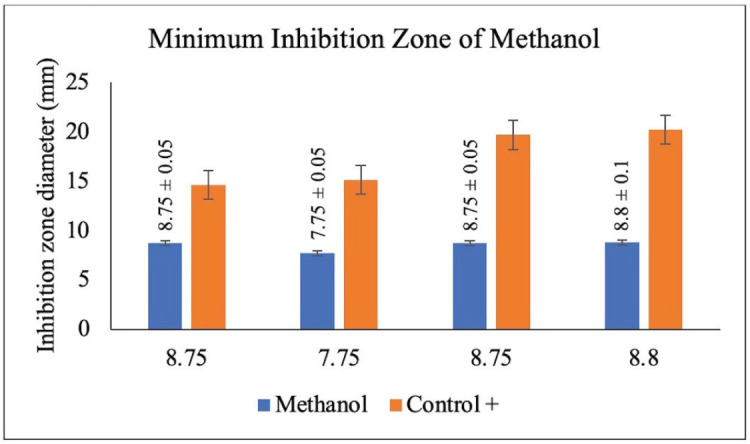
The results of the antibacterial activity test using methanol fraction of red ginger.

**Figure 3.**
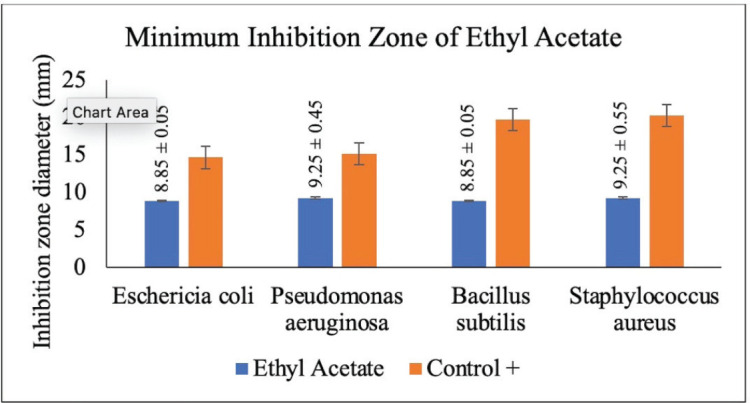
The results of the antibacterial activity test using ethyl acetate fraction of red ginger.

**Figure 4.**
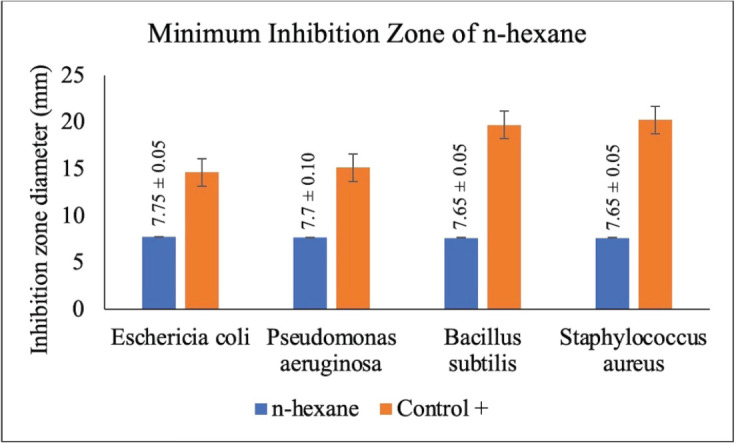
The results of the antibacterial activity test using the n-hexane fraction of red ginger.

The antibacterial activity test on each fraction of red ginger has been completed, and it was discovered that the extract of red ginger has moderate activity in inhibiting the growth of tested bacteria. The inhibition of the growth of the tested bacteria can be seen in Table 1, where the ethyl acetate fraction was found to have the best inhibition power compared to methanol and n-hexane. The ability of the ethyl acetate fraction to inhibit the growth of bacteria can be seen from the large size of the inhibition zone produced for each tested bacterium. This was comparable to the size of the inhibition zone produced on the trial test. The ability of the ethyl acetate fraction to inhibit bacterial growth is associated with the secondary metabolites such as triterpenoids, phenolics, and flavonoids contained in the fraction. The secondary metabolites in the red ginger rhizome can obstruct the growth of bacteria. This is in line with a study conducted by Prakosa et al. [37], who found that phenolics, flavonoids, alkaloids, and saponins have actively contributed to inhibiting and damaging the growth of *Aeromonas hydrophila*. The ability of red ginger extract to inhibit the growth of tested bacteria can be seen in Figures 2–4.

### MIC activity test

To observe the activity of the minimum inhibitory concentration, a turbidimetry observation or visual turbidity observation was carried out. From the minimum inhibitory concentration activity test, the minimum concentration of each fraction (methanol, ethyl acetate, and n-hexane) of red ginger required to inhibit bacteria growth was identified. This observation is proportional to the bacteria growth inhibition test using the disc diffusion method, where ethyl acetate still has a good ability to inhibit the bacteria growth compared to the other two fractions. This observation on the minimum inhibitory concentration of each extract was done after 24 h of incubation by observing the turbidity level of the tested solution on each microplate. The results showed that the extract of ethyl acetate effectively inhibits the growth of Gram-positive and Gram-negative bacteria. *B. subtilis* bacteria were extremely resistant to ethyl acetate extract, with an inhibition zone value of 8.85 ± 0.05 at a concentration of 3.125 mg/ml ethyl acetate. Moreover, the *S. aureus* bacteria growth was inhibited in the inhibition zone ranging around 9.25 ± 0.05 with a concentration value of 6.25 mg/ml of ethyl acetate extract.

While for the Gram-positive bacteria (*E. coli* and *P. aeruginosa*), their growth was inhibited in the inhibition zone with a range of 8.85 ± 0.05–9.25 ± 0.05 with a concentration of ethyl acetate at 6.25 mg/ml. This minimum inhibitory concentration value discloses that the antibacterial activity of ethyl acetate is stronger than that of methanol and n-hexane fractions [38]. The comparison of the minimum inhibitory concentrations of the three fractions of red ginger can be seen in Table 7. The result of the study shows that the minimum concentration of each fraction to inhibit bacterial growth in a sequence is ethyl acetate > methanol > n-hexane.

### MBC activity test

The MBC test on red ginger extract aims to observe the ability of each extract to kill the tested bacteria. The tested bacteria used in this study were gram-positive, namely *B. subtilis* and *S. aureus*, and gram-negative, such as *E. coli* and *P. aeruginosa*. The efficacy of each fraction (methanol, ethyl acetate, and n-hexane) in killing bacteria would be seen after 24 h of incubation. Based on the observation, it was identified that the ethyl acetate fraction has a stronger capability than methanol and that n-hexane has the lowest killing power. The value was obtained after observing the bacteria growth on media that had been incubated for 24 h, where the bacteria were plated at the lowest concentration to analyze the MBC score through the turbidity level of the solution produced. The MBC value of the extract of ethyl acetate ranges from 6.25 to 12.5 mg/ml for the four tested bacteria. The relatively low score of the extract of the ethyl acetate fraction is believed to enable it to inhibit the growth of tested bacteria [39]. The detailed score of the MBC of the three extracts can be seen in Table 8.

**Table 7.** The comparison of the minimum inhibitory concentration of each fraction toward four tested bacteria.

Minimum inhibitory concentration (mg/ml)				
	*E. coli*	*P. aeruginosa*	*B. subtilis*	*S. aureus*
Methanol	12.50	18.75	25.00	25.00
Ethyl acetate	6.25	6.25	3.125	6.25
n-hexane	62.50	87.50	87.50	100.00
Control +	0.625	0.625	0.625	0.625
Control -	100.00	100.00	100.00	100.00
Documentation	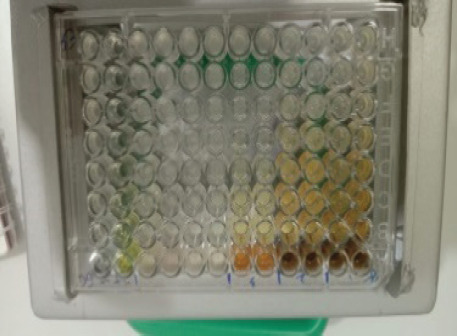	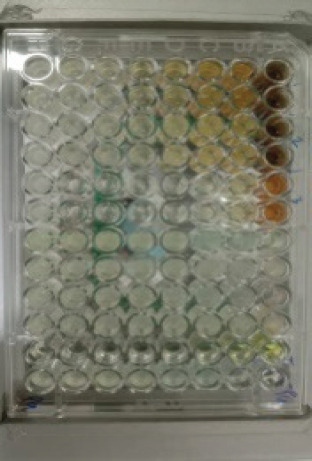	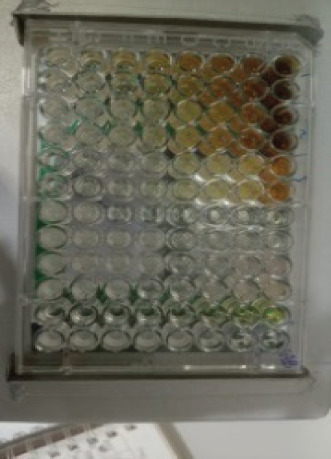	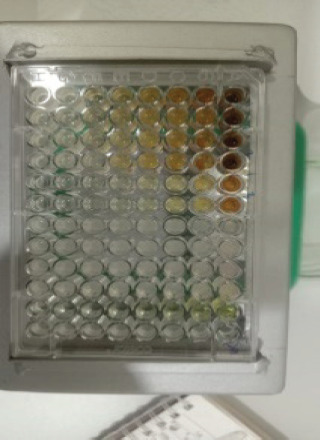
	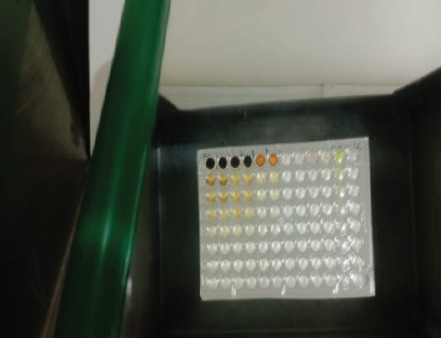	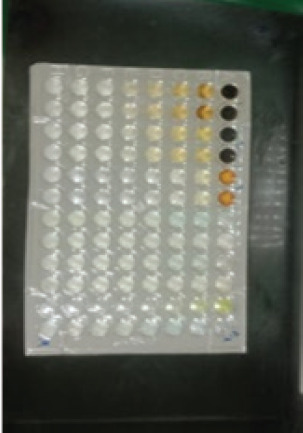	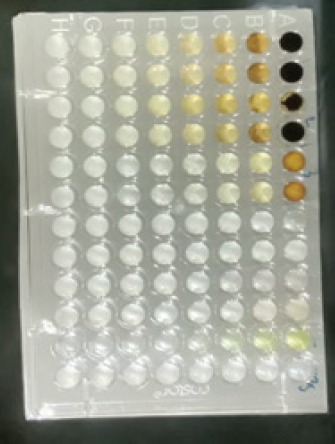	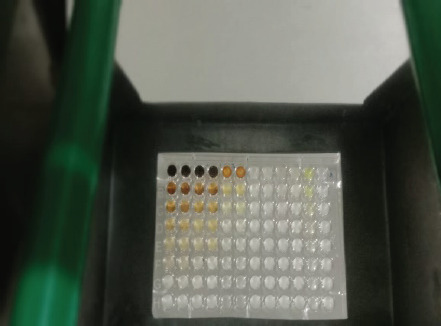

*Note:* The Minimum Inhibitory Concentration test was conducted in the Microbiology Laboratory, Vaccine and Drug Research Centre, National Research and Innovation Agency, 2022.

**Table 8.** The MBC of the three extracts of red ginger against the tested bacteria.

MBC (mg/ml)				
Sample	Bacteria/concentration			
	*E. coli*	*P. aeruginosa*	*B. subtilis*	*S. aureus*
Methanol	> 18.75	> 18.75	> 25.00	> 25.00
Ethyl acetate	> 6.25	> 12.50	> 6.25	> 6.25
n-hexane	> 87.50	> 87.50	> 125.0	> 137.5
Control +	> 0.625	> 0.625	> 0.625	> 0.625
Control -	> 100	> 100	> 100	> 100

*Note:* The MBC test of the three fractions was conducted in the Microbiology Laboratory, Vaccine and Drug Research Centre, National Research and Innovation Agency, 2022.

## Conclusion

The red ginger extract fractionated using three types of solvent with different polarity levels reveals information regarding the total number of secondary metabolites such as phenolics, flavonoids, alkaloids, coumarins, triterpenoids, steroids, and beta-carotene groups in the n-hexane fraction. Phenolics and flavonoids in ethyl acetate have good activity in capturing free radicals that are produced from DPPH, with an IC_50_ value of 49.2610 mg/l. Meanwhile, the antioxidant activity of methanol and n-hexane is relatively low, at 146.648 and 300.865 mg/l, respectively. The antibacterial activity of the three fractions of red ginger shows ethyl acetate as the fraction possessing the most effective inhibition compared to methanol and n-hexane, respectively. Furthermore, the fractions of ethyl acetate and methanol have good activities in inhibiting and eradicating the growth of gram-positive bacteria such as *B. subtilis* and *S. aureus*, and gram-negative bacteria such as *E. coli* and *P. aeruginosa*. The n-hexane fraction, on the other hand, shows moderate bacterial activity to inhibit bacterial growth. The value of the minimum inhibition concentration in this study was higher than the control positive tetracyclin. However, its use is at a safe concentration level due to the nature of the samples, which come from natural elements, not synthetic chemicals.

## List of Abbreviations

CFU, colony forming units; DPPH, 2,2-diphenyl-1-picryl-hydrazine-hydrate; gm, gram; HCl, hydrochloride; h, hours; IC_50_, inhibitory concentration; MBC, minimum bactericidal concentration; mg/l, milligram per liter; mg/ml, milligram per milliliter; mg GA/extract, milli gram gallic acid per extract; MIC, minimum inhibitory concentration; ml, milliliter; Qu/extract, quercetin per extract; *R*^2^, correlation coefficient value.
